# Contagious Ovine Digital Dermatitis: A Novel Bacterial Etiology and Lesion Pathogenesis

**DOI:** 10.3389/fvets.2021.722461

**Published:** 2021-09-23

**Authors:** Gareth J. Staton, Joseph W. Angell, Dai Grove-White, Simon R. Clegg, Stuart D. Carter, Nicholas J. Evans, Jennifer S. Duncan

**Affiliations:** ^1^Department of Infection Biology & Microbiomes, Institute of Infection, Veterinary and Ecological Sciences, The University of Liverpool, Neston, United Kingdom; ^2^Wern Vets CYF, Department of Research and Innovation, Unit 11, Lon Parcwr Industrial Estate, Ruthin, United Kingdom; ^3^Department of Livestock and One Health, Institute of Infection, Veterinary and Ecological Sciences, University of Liverpool, Neston, United Kingdom; ^4^School of Life Sciences, College of Science, University of Lincoln, Lincoln, United Kingdom

**Keywords:** sheep, lameness, CODD, footrot, *Treponema medium*, *Treponema phagedenis*, *Treponema pedis*, *Dichelobacter nodosus*

## Abstract

Contagious ovine digital dermatitis (CODD) is a severe and common infectious foot disease of sheep and a significant animal welfare issue for the sheep industry in the UK and some European countries. The etiology and pathogenesis of the disease are incompletely understood. In this longitudinal, experimental study, CODD was induced in 18 sheep, and for the first time, the clinical lesion development and associated microbiological changes in CODD affected feet are described over time, resulting in a completely new understanding of the etiopathogenesis of CODD. The majority of CODD lesions (83.9%) arose from pre-existing interdigital dermatitis (ID) and/or footrot (FR) lesions. All stages of foot disease were associated with high levels of poly-bacterial colonization with five pathogens, which were detected by quantitative PCR (qPCR): *Treponema medium, Treponema phagedenis, Treponema pedis, Dichelobacter nodosus*, and *Fusobacterium necrophorum*. Temporal colonization patterns showed a trend for early colonization by *T. phagedenis*, followed by *F. necrophorum* and *D. nodosus, T. medium*, and then *T. pedis, D. nodosus* was present at significantly higher predicted mean log_10_ genome copy numbers in FR lesions compared to both ID and CODD, while *Treponema* species were significantly higher in CODD and FR lesions compared to ID lesions (*p* < 0.001). Treatment of CODD-affected sheep with two doses of 10 mg/kg long acting amoxicillin resulted in a 91.7% clinical cure rate by 3 weeks post-treatment; however, a bacteriological cure was not established for all CODD-affected feet. The study found that in an infected flock, healthy feet, healed CODD feet, and treated CODD feet can be colonized by some or all of the five pathogens associated with CODD and therefore could be a source of continued infection in flocks. The study is an experimental study, and the findings require validation in field CODD cases. However, it does provide a new understanding of the etiopathogenesis of CODD and further supportive evidence for the importance of current advice on the control of CODD; namely, ensuring optimum flock control of footrot and prompt isolation and effective treatment of clinical cases.

## Introduction

Contagious ovine digital dermatitis (CODD) is a relatively new infectious foot disease of sheep, first recorded in the UK in 1997 (1). It is now widespread in the UK, affecting an estimated 35–58% of sheep flocks (2, 3) and has also been reported in Ireland (4), Germany (5), and Sweden (personal comm.). CODD is the most severe form of sheep lameness recorded (6), and coupled with challenges around disease control in infected flocks (7), CODD has a substantially negative impact on sheep welfare and is a priority issue for the sheep industry.

**Figure 1:**
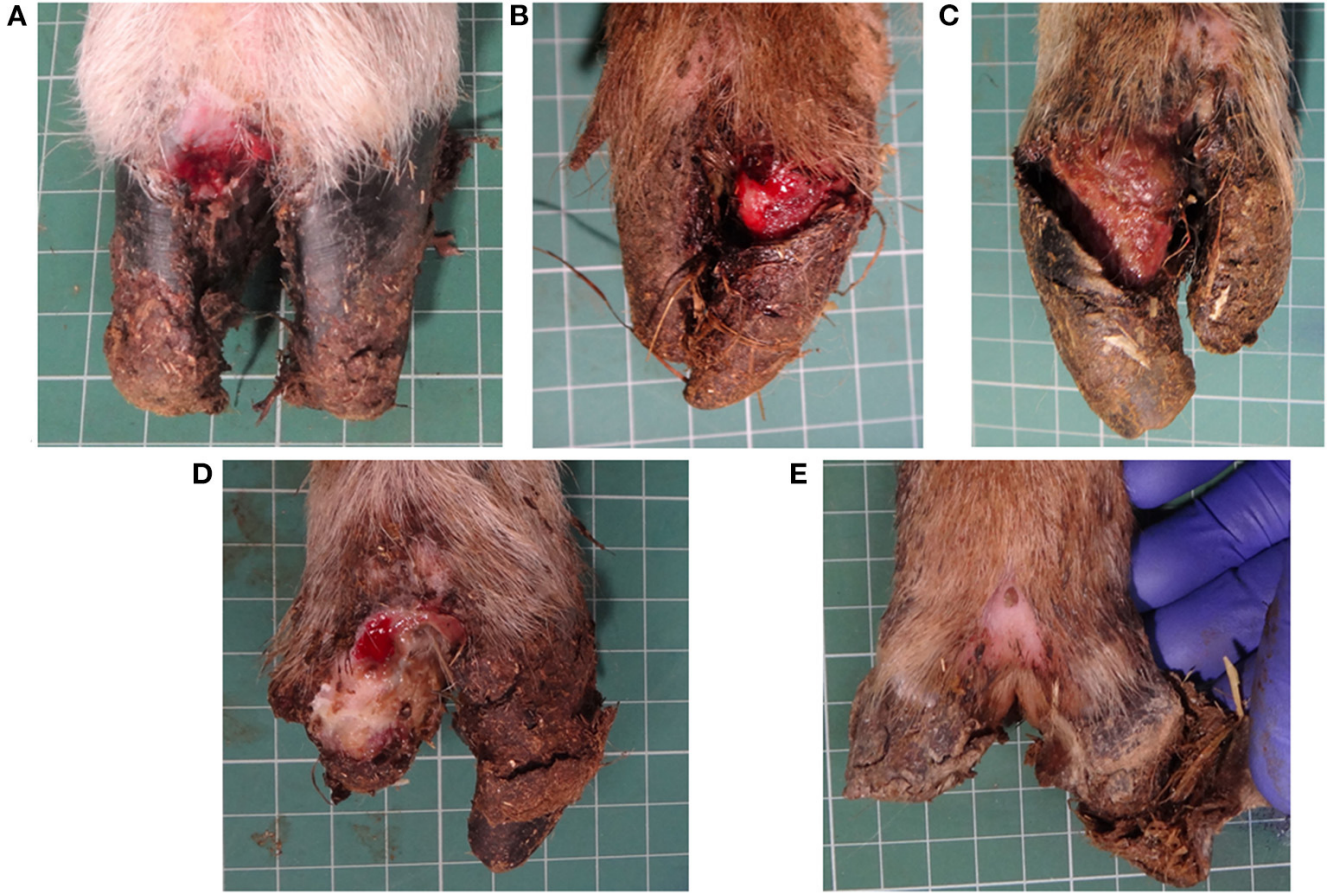
CODD G#1 **(A)**, CODD G#2 **(B)**, CODD G#3 **(C)**, CODD G#4 **(D)**, and CODD G#5 **(E)**.

The severity of lameness in CODD-affected sheep is a consequence of the extensive foot pathology caused by the disease (Figure 1). CODD is a progressive infectious foot disease, which begins with an inflammatory lesion on the dorsal coronary band of the hoof and culminates in avulsion of the entire hoof capsule leaving highly sensitive underlying hoof lamellae tissue exposed (Figure 1). Furthermore, radiographic investigation of affected feet revealed periosteal inflammatory changes in the pedal bone in most stages of the disease, indicating extensive internal damage to the structure of the sheep's foot and a further source of pain and functional compromise (6). Histopathological examinations of early stage lesions are described as lymphoplasmacytic infiltration of the distal digital skin with suppurative coronitis and intracorneal pustules; in the more advanced stages of disease, there is complete separation of the dorsal wall of the hoof with a necrotizing and fibrinosuppurative exudate and dermatitis (8).

The etiology of CODD is considered to be bacterial. From the earliest microbiology studies, species of the *Treponema* genus of bacteria have been associated with CODD lesions (4, 9–11); in particular, one of the three treponemal species, which are considered causal for bovine digital dermatitis (BDD), are consistently found in CODD lesions. These are *Treponema medium, Treponema phagedenis*, and *Treponema pedis* (4, 8, 12, 13). Therefore, it is hypothesized that these three digital dermatitis (DD)-associated treponemes may have crossed species from cattle to sheep to initiate the emergence of CODD in sheep (14). Immunohistochemical analysis of CODD lesions also clearly demonstrate large numbers of unspecified *Treponema*-like organisms closely associated with the histopathological changes of CODD lesions, thus providing further evidence for the role of treponeme bacteria in the etiology of CODD (8).

However, a single pathogen etiology for CODD has not been wholly established. Other bacterial species have also been repeatedly isolated and identified in CODD lesions, namely, *D. nodosus* and *F. necrophorum* (12, 15). *Dichelobacter nodosus* is the causal agent of footrot in sheep, while *F. necrophorum* is considered a secondary invading pathogen in footrot lesions (16, 17) and other diseases. Furthermore, epidemiological evidence has repeatedly demonstrated strong associations between footrot and CODD (2, 18), and vaccinating animals against *D. nodosus* does provide some protection against CODD infection (19). Therefore, it is possible that either of these two known foot pathogens may play a role in the pathogenesis of CODD. They may indeed be the primary pathogens of CODD; they may provide initiating skin/horn damage allowing secondary treponemal invasion, or they may be secondary pathogens of already established CODD lesions. They could also be contaminants of no pathogenic significance at all. Consequently, the precise etiology of CODD remains unproven.

There are several important limiting factors to previous research on the etiology of CODD, which need to be overcome in order to clarify its etiology. These include the following:

1. *Sampling strategy*: To date, studies so far have employed a cross-sectional sampling study design. Such methods provide information on the presence of bacteria in foot lesions at a single time point. However, they provide limited evidence of disease causality in terms of bacterial initiation and lesion progression.
2. *Culture method bias*: It is well-recognized that the culture method used in a bacteriological study will strongly influence the bacterial organisms identified from a sample, meaning that many bacteria that may well be significant in a disease process may not be detected (20). Bacteria associated with ovine foot disease are noted for their particularly fastidious nature, and therefore, false negative culture results are likely (15). Despite these limitations, culturing bacteria from a sample does indicate that the organisms are present and viable in the tissue, which is one of Koch's original postulates for determining disease causality (21). Furthermore, isolating bacteria from lesions allows for further biochemical and molecular characterization of the organisms (22).
3. *Interpretation of PCR data*: Routine diagnostic PCR data are limited to providing binary information on the presence or absence of a bacterial species in a sample. Quantitative molecular data on whether the organism is multiplying in a lesion is helpful in supporting causality and can help to distinguish between the inevitable environmental contamination of bacteriological samples collected from sheep's feet and pathogens that are actively multiplying in a lesion.

Therefore, bearing these previous study limitations in mind, the aim of the current study was to investigate the bacterial etiology of CODD in a longitudinal, experimental study of naturally occurring CODD lesions; employing a quantitative PCR methodology to determine the temporal associations of previously identified bacterial species of *T. medium, T. phagedenis, T. pedis, D. nodosus*, and *F. necrophorum* in the etiology of CODD.

## Materials and Methods

### Experimental Design

The project was carried out under UK Animal Scientific Procedure Act (ASPA) 1986, Home Office Project License PPL 708756, and University of Liverpool Ethics VREC417. The experimental study was supervised at all times by a named Animal Care and Welfare Officer and a team of three veterinary surgeons. The reporting of the experiment is in accordance with the ARRIVE guidelines [(23); Supplementary Material 1].

The study design was an observational study of an experimentally induced outbreak of CODD in housed sheep whereby 30 healthy, 18-month-old, Texel cross-bred ewes were housed with 10 sheep of mixed age and breed, affected by CODD. Inclusion criteria for healthy ewes were acquisition from a single flock with no known history of CODD and same sex, breed, and age. Inclusion criteria for infected sheep were that they were sourced from farms with a history of CODD in the flock and should have a confirmed veterinary diagnosis of an active, untreated CODD lesion on one foot. At study start, all infected sheep were PCR positive (13) for at least one of the hypothesized causal pathogens of CODD (*T. medium, T. phagedenis*, and *T. pedis)*. Sample size power calculations were not made for due to lack of data on expected variation in the microbiological consortium; however, sample sizes were consistent with other similar studies (17, 24). The observational design of the experiment meant that it did not require blinding or randomizing.

The sheep were housed in a Home Office Designated Building (according to UK Animal Scientific Procedures Act, Code of Practice for Care and Accommodation of Animals) on deep litter straw bedding at a stocking rate of 1.9 m^2^/sheep. Sheep were fed a maintenance ration of *ad libitum* hay. A footbath was placed under the feed racks, which contained damp straw, water, and contaminated hoof clippings from a CODD-infected farm to simulate naturally occurring risk factors for CODD. Sheep welfare was monitored by daily inspection of demeanor and feed intake, twice weekly locomotion (25) and body condition scoring (26), and weekly veterinary clinical examination. Humane endpoints were set (inappetence, recumbency, or non-weight bearing lameness on any limb), and if an animal reached these predetermined points, the animal was withdrawn from study. When half of the sheep in the flock had developed CODD lesions, all sheep with any foot lesion were treated with two doses, 48 h apart, of a long acting amoxicillin (Betamox LA 150 mg/ml, Norbrook, Northern Ireland, UK) at a dose rate of 10 mg/kg administered by intramuscular injection. In addition, all bedding was removed from the housing, all flooring and fitments cleaned by power washing with water and disinfectant (FAM 30, Evans Vanodine plc, Preston, UK), and fresh straw bedding provided.

### Animal Sampling

At the start of the project and during every week of the study, the following data and samples were collected from each sheep: a locomotion score (25), a body condition score (26), a foot lesion score of each foot (6), and foot skin swab (Copan, Italy) from each foot. When a foot lesion was present, a swab was applied to the entire surface of the visible lesion. Collected swabs were immediately stored at −80°C until DNA extraction. Animal metadata were stored on an Access Database (Microsoft, Redmond, WA, USA).

### Foot Lesion Classification

**Figure 2:**
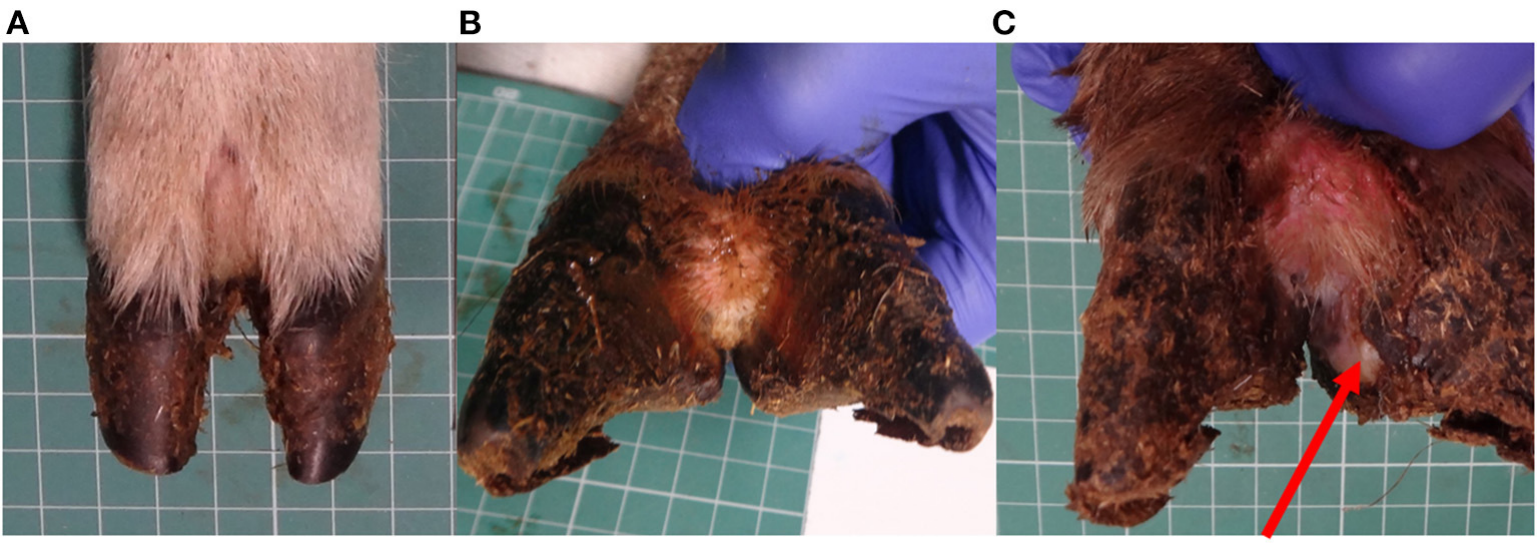
Healthy sheep's foot **(A)**, ID lesion **(B)**, and FR lesion **(C)**, arrow pointing to under run horn tissue.

All locomotion and lesion scoring observations were performed by one of two experienced observers (JA and JD). Sheep were locomotion scored using a 4-point ordinal locomotion scoring system that measured the degree of lameness exhibited by the sheep from sound (score 0), mild (score 1), moderate (score 2), and severe (score 3) (25). Foot lesions were classified on the basis of their clinical appearance as interdigital dermatitis (ID) or footrot (FR) as per published descriptions (27) and CODD (6). ID was defined as any degree of inflammation of the interdigital skin only, while FR was defined as the presence of underrunning of the horn of the heel and sole (Figure 2). CODD lesions were graded (G#) as follows: G#1 is described as a focally extensive erosive or ulcerative lesion affecting the digital skin and coronary band; a G#2 lesion is reported as a separation between the hoof capsule and the hoof lamellae affecting up to 50% of the dorsal and abaxial hoof wall; a G#3 lesion is described as separation between the hoof capsule and lamellae affecting >50% of the dorsal and abaxial hoof wall; a G#4 lesion is described where there is evidence of horn regrowth on the surface of the lamellae but not over the entire surface (lamellar tissue remains exposed); in G#5 lesions, horn regrowth is apparent over the entire surface of the lamellae, although the horn surface maybe smooth and distorted by circumferential ridges and the affected digit may be wider and shortened in comparison to the unaffected digit (Figure 1).

In some cases, feet could be considered to have features of both footrot and CODD, or interdigital dermatitis (scald) and CODD. In these cases, the combination of lesions was recorded.

### Isolation of Foot Swab DNA

Genomic DNA was isolated from foot swab samples using the DNeasy Blood and Tissue Kit (Qiagen, Manchester, UK), according to the manufacturer's instructions, as described previously (28). Extracted DNA was stored at −80°C until analysis.

### qPCR

#### Primer and Probe Design

Previously described Taqman qPCR primer and probes targeting a 61-bp sequence within the RNA polymerase sigma-70 factor gene (rpoD) of *Dichelobacter nodosus* (29) and an 86-bp sequence within the β-subunit of RNA polymerase gene (*rpoB*) of *Fusobacterium necrophorum* (17) were employed. In addition, three novel Taqman qPCR assays were designed to individually target 254, 234, and 247 bp sequences within the Recombinase A (RecA) genes of *T. medium* (accession number CP027017), *T. phagedenis* (accession number CP027018), and *T. pedis* (accession number CP045760), respectively. The RecA gene was selected because of its singular occurrence in the genomes of *Treponema* spp. and its relatively low within-species diversity across a global panel of digital dermatitis-associated treponeme isolates (30). RecA gene sequences were extracted from the sequenced genomes, aligned with all available *recA* alleles from the previous multilocus sequence typing study and subjected to primer design using Mega X (31) and Primer 3 (32). The quality of the primer and probe sequences were analyzed using OligoCalc (33). The primer and probe sequences for the *T. medium, T. phagedenis*, and *T. pedis* qPCR assays are shown in Table 1. All probes were labeled with the fluorophore, 6-carboxyl-fluorescein (FAM), at the 5′-end and the non-fluorescent Black Berry Quencher (BBQ) at the 3′-end. Primers and probes were synthesized and purified commercially (TIB MOLBIOL, GmbH, Berlin, Germany).

**Table 1:** Primer and probe oligonucleotide sequences used in the Taqman qPCR assays targeting the *RecA* gene of *T. medium, T. phagedenis*, and *T. pedis*.

**Target species**	**Oligonucleotide**	**Sequence (5^**′**^-3^**′**^)**
*T. medium*	Forward primer	CTACAAATCGAAAAGGAGTTTGGA
	Reverse primer	GGCATGTTCGGCATCCAC
	Probe	TAGAATTATCGAAATATTCGGCCCAGA
*T. phagedenis*	Forward primer	GCCTTCAAATCGAAAAACAATTC
	Reverse primer	GCCGCAATGCCGCCGCG
	Probe	TAGATGAGGCACTGGGAATCGG
*T. pedis*	Forward primer	AAATTGAAAAACAATTCGGACAG
	Reverse primer	GTGTTCGGCATCTATAAAAGCC
	Probe	ATACCCCAGAGGCCGTATTATCGAG

#### Standard Curve Preparation

A 61-bp *rpoD* gene fragment from *D. nodosus* strain VCS1703A was commercially synthesized and inserted into a pGM plasmid (GeneMill, University of Liverpool, UK). The plasmid was propagated in Top10 *Escherichia coli* (Life Technologies, Paisley, UK) according to the manufacturer's instructions and purified using the Plasmid Mini Kit (Qiagen, Manchester, UK). *Fusobacterium necrophorum* subs. *funduliforme*, previously isolated from a bovine digital dermatitis lesion by our laboratory, was grown in oral treponeme enrichment broth (OTEB, Anaerobe Systems, Morgan Hill, CA, USA). *T. medium* T19 was cultured in OTEB supplemented with 10% (v/v) rabbit serum, while *T. phagedenis* T320A and *T. pedis* T3552B^T^ were cultured in OTEB supplemented with 10 % (v/v) fetal calf serum as previously described (34). Chromosomal DNA was extracted using the Wizard kit (Promega, Southampton, UK) according to manufacturer's instructions and quantified using the Nanodrop ND-2000 spectrophotometer (Thermo Fisher Scientific, Loughborough, UK). For each bacterial DNA preparation, genome copy numbers were calculated using the following equation: number of copies of DNA template per μl = (DNA concentration (ng/μl) × Avogadro's number)/[length of template (bp) × conversion factor to ng × average weight of a base pair (Da)]. Serial dilutions of the purified bacterial DNA were subsequently prepared in 10 mM Tris-Cl, pH 8.5, to yield estimated genome copy number of 3.25 × 10^6^ to 3.25 × 10^−1^, 1.03 × 10^6^ to 1.03 × 10^−1^, 2.66 × 10^6^ to 2.66 × 10^−1^, 6.31 × 10^8^ to 6.31 × 10^−1^, and 4.37 × 10^6^ to 4.37 × 10^−1^ for *T. medium, T. phagedenis, T. pedis, D. nodosus*, and *F. necrophorum*, respectively.

#### qPCR Cycling Conditions

All qPCR assays were performed on the 7900HT Fast Real-Time PCR System (Applied Biosystems, Foster City, CA, USA), and each 25 μl reaction comprised of the following: 5 μl 5 × HOT FIREPol® Probe qPCR Mix Plus (ROX) (Solis Biodyne, Tartu, Estonia), 1 μl (0.4 μM) forward primer, 1 μl (0.4 μM) reverse primer, 0.625 μl (0.25 μM) probe, 16.375 μl PCR-grade water, and 1 μl of DNA template. All reactions were performed in triplicate. The *F. necrophorum* (17) and *D. nodosus* (29) qPCR assays were performed using the cycling conditions previously described. Optimal qPCR cycling conditions for the three *Treponema* qPCR assays were empirically determined and comprised of a single activation step of 95°C for 12 min followed by 40 cycles of 95°C for 15 s and 61°C for 15 s and 72°C for 30 s. Analytical specificity and sensitivity were empirically defined for each assay. The baseline fluorescence signal was automatically calculated from the first 3–15 qPCR cycles. Where no increase in fluorescence signal was detected after 40 cycles, the bacterial load was classified as “undetectable,” i.e., below the limit of detection.

#### qPCR Analytical Specificities and Limits of Detection

Analytical specificity of the *D. nodosus* and *F. necrophorum* qPCR assays have been previously described (17, 29). The specificity of the *F. necrophorum rpoB* assay was verified by analyzing the genomic DNA of *Fusobacterium varium*, a species closely related to *F. necrophorum*, while the genomic DNA of *F. necrophorum* subsp. *necrophorum* (DSM21784) (DSMZ, Germany) was used as a positive control. The specificity of the *D. nodosus rpoD* assay was verified by analyzing the genomic DNA of the two closest relatives of *D. nodosus*, namely, *Suttonella indologenes* (DSM8309) and *Cardiobacterium hominis* (DSM8339), while using the genomic DNA of *D. nodosus* (DSM23057) as a positive control. The specificities of the three novel pathogenic *Treponema* qPCR assays were individually established by analyzing the genomic DNA of *T. medium, T. phagedenis*, and *T. pedis*, to confirm the absence of cross-reactivity between the three assays, and by analyzing the genomic DNA of two commensal treponemes, namely, *T. ruminis* (DSM 103462) and *T. rectale* (DSM 103679). The lower limits of detection for the *T. medium, T. phagedenis, T. pedis, D. nodosus*, and *F. necrophorum* assays were 32.5, 10.3, 26.6, 63.1, and 43.7 genome copies/μl template, respectively; below these concentrations, DNA detection failed. The calibration standards for the *T. medium, T. phagedenis, T. pedis, D. nodosus*, and *F. necrophorum* assays generated *R*^2^-values of >0.99 and mean slopes of −3.5 (SEM ± 0.04), −3.5 (SEM ± 0.01), −3.5 (SEM ± 0.07), −3.4 (SEM ± 0.07), and −3.7 (SEM ± 0.06), respectively, indicating that the amplification efficiency of these assays was >85%.

Each sample assay was performed in triplicate, and the mean genome copy number (MGCN) was calculated from the three assay results.

### Statistical Analysis

All data were recorded in Access database (Microsoft, Redmond, WA, USA) spreadsheets. Following the manual cleaning, data were exported to Stata 16 (StataCorp, College Station, TX, USA) for further cleaning, data checking, appropriate recoding where required, and statistical analysis. Binary variables based on MGCN values were produced for all bacterial species using the respective lower limits of detection defined above, with values above these values indicating presence of the bacterial species. MGCN data were log_10_ transformed for analysis. Mean values and proportions were estimated with 95% confidence intervals where appropriate. Comparison of mean log_10_ MGCN values were performed using one-way ANOVA with a Bonferroni correction for multiple comparisons. Comparison of proportional data were performed using Fisher's exact test. Survival analysis with production of Kaplan–Meier plots was performed to investigate time to first recording of a specific foot lesion and time to first colonization by a specific bacterial species. Mixed effects linear regression models with random effects at sheep and foot level were fitted to investigate associations between lesion or CODD grade and log_10_ MGCN. Predicted marginal mean estimates were obtained from models and presented graphically.

## Results

### Clinical Findings

Thirty, non-lame experimental sheep were recruited to the study. The duration of the study was 26 weeks. A total of 2,392 recordings of digit lesions were made during the study period. During the study period, 24 sheep were recorded as having a foot lesion associated with lameness in one or more feet on at least one occasion with only 6 sheep showing no lesions throughout the study period. A total of 59 feet (49.2%) were recorded as having a lesion on at least one occasion. The distribution of lesions recorded at foot level is as follows: no lesions (86.29%), ID (3.64% of recordings), FR (2.68% of recordings), CODD (5.85% of recordings), and other lesions (1.55% of recordings).

**Figure 3:**
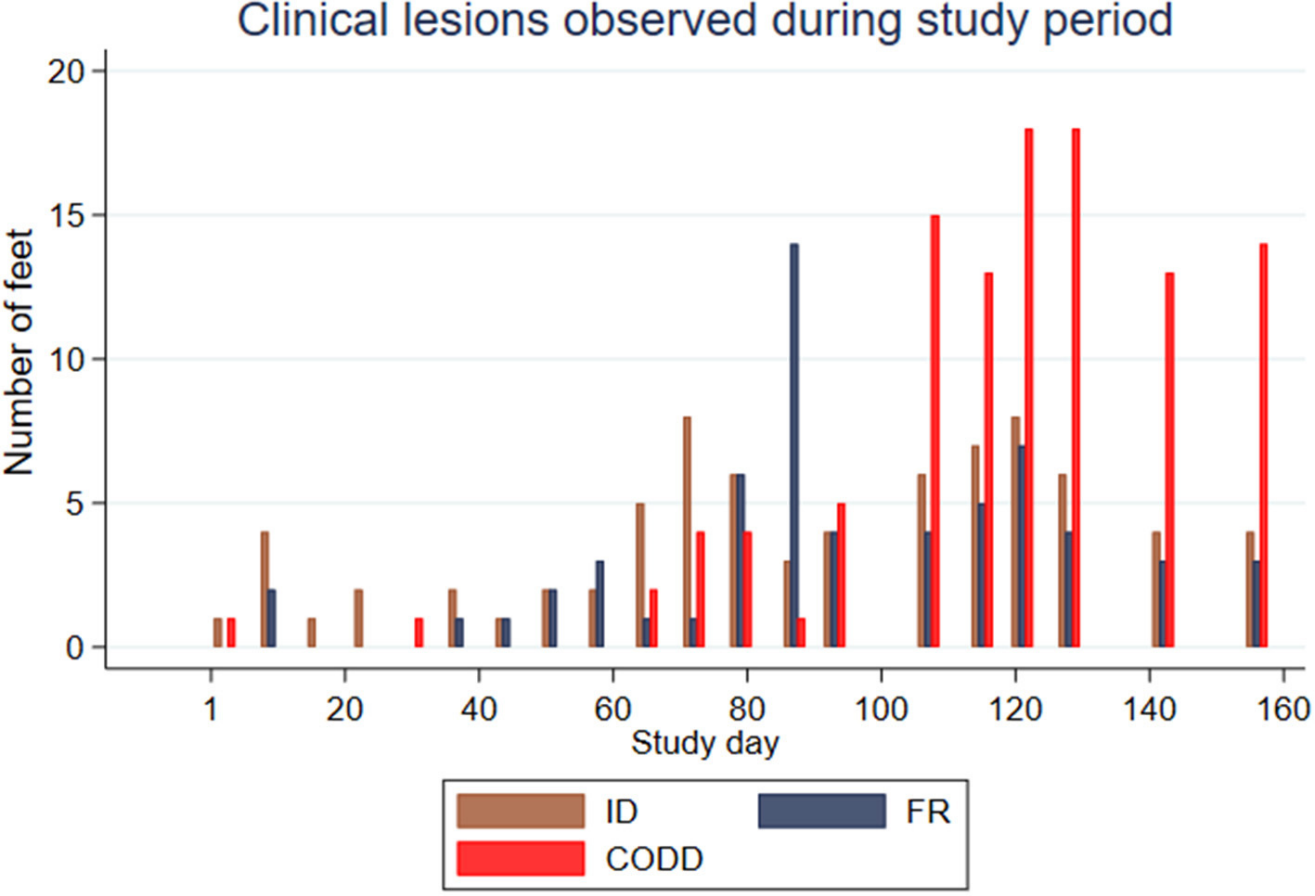
Clinical lesions observed during study period for the experimental sheep (*n* = 30 sheep, *n* = 120 feet).

Feet were affected by different lesions throughout the study period, but there appeared to be a broad temporal trend in the distribution of lesions, with ID and FR lesions representing the greatest proportion of lesions initially with an increasing proportion of CODD lesions observed as the study progressed (Figure 3).

**Figure 4:**
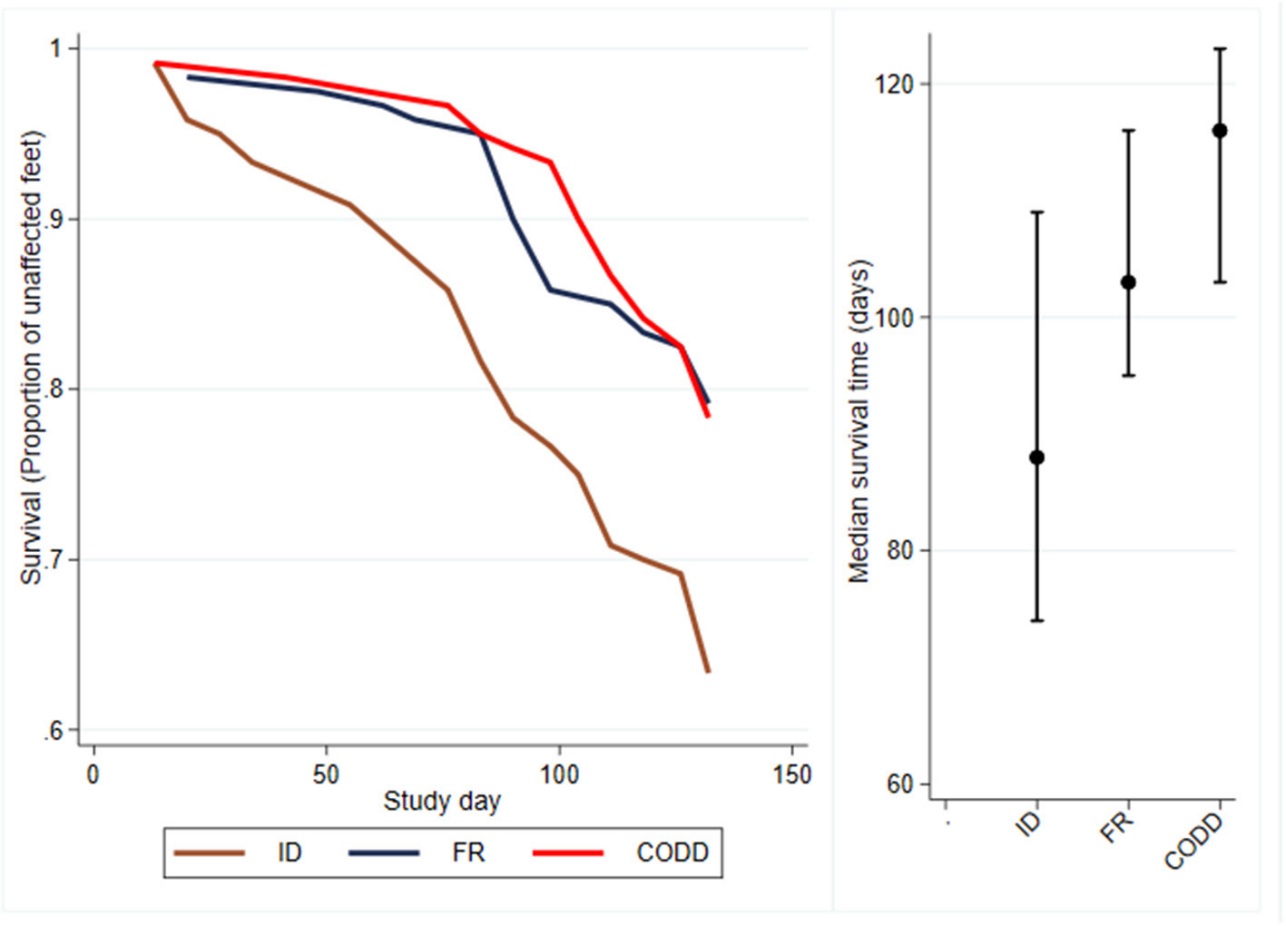
Kaplan–Meier survival curves and median times to first observation of a clinical lesion for the experimental sheep (*n* = 30 sheep, *n* = 120 feet).

Survival analysis was performed to investigate time to first recording of a lesion in a foot and Kaplan–Meier survival curves plotted together with median survival times (95% CI) (Figure 4). These suggest that ID lesions were first recorded (median survival time, 88 days; 95% CI 74–109 days), followed by FR (median survival time, 103 days; 95% CI, 95–116 days), then CODD (median survival time, 116 days; 95% CI, 103–123 days). The confidence intervals are large and overlap.

There was considerable heterogeneity in lesions recorded with multiple different lesions recorded on the same foot during the study period. For analysis purposes, CODD was assumed to be the final outcome in terms of progression severity, and feet were then classified according to the final outcome. ID was recorded as the final outcome in 19 feet (15.8%) and FR in 9 feet (7.5%), and CODD was recorded as the final outcome lesion in 31 feet (25.8%).

The majority of CODD lesions appeared to arise following previous ID and/or FR lesions. Only 5/31 (16%) of CODD lesions developed *de novo*, while 21/31 (64%) were preceded by lesions of FR (with or without ID), and 6/31 (19%) were preceded by lesions of ID only.

A total of 31 feet were recorded with a CODD lesion on at least one occasion with the following CODD grade distribution: G#1, *n* = 25 feet; G#2, *n* = 14 feet; G#3, *n* = 7; G#4, *n* = 6; G#5, *n* = 16. While the majority of feet with CODD lesions had prior lesions of either ID (*n* = 6) or FR (*n* = 3) or both ID and FR (*n* = 17), CODD arose *de novo* in five feet (16%). It is worthy of note that of the five feet in which CODD arose *de novo*, all were recorded with G#1 lesions, of which only one progressed directly to G#5, while the remaining four feet resolved spontaneously without further progression. Overall, this suggests that CODD lesions arising *de novo* did not progress further to lesions of “active CODD” but either resolved spontaneously or were recorded as G#5 (“healed lesions”).

Of the 26 feet that developed CODD subsequent to either ID, FR, or FR and ID, there was a trend for lesions to progress in severity through the CODD disease process (CODD G#1–G#5). However, there was temporal “waxing and waning” in lesion severity, although the overall trend was one of increasing lesion severity.

There were 12 CODD lesions (G#1–G#4) present in nine sheep at the time of treatment of the flock. The treatment was two doses, 48 h apart, of a long acting amoxicillin (Betamox LA 150 mg/ml, Norbrook, Northern Ireland, UK) at dose rate of 10 mg/kg by intramuscular injection together with environmental decontamination. One week following completion of treatment, 100% of the CODD foot lesions had resolved to CODD 5 or healthy status. However, by 3 weeks post-treatment, one CODD foot lesion (8.33%) was noted to have clinically recurred (CODD G#1).

### Microbiological Findings

#### Microbiology of ID, FR, and CODD

**Figure 5:**
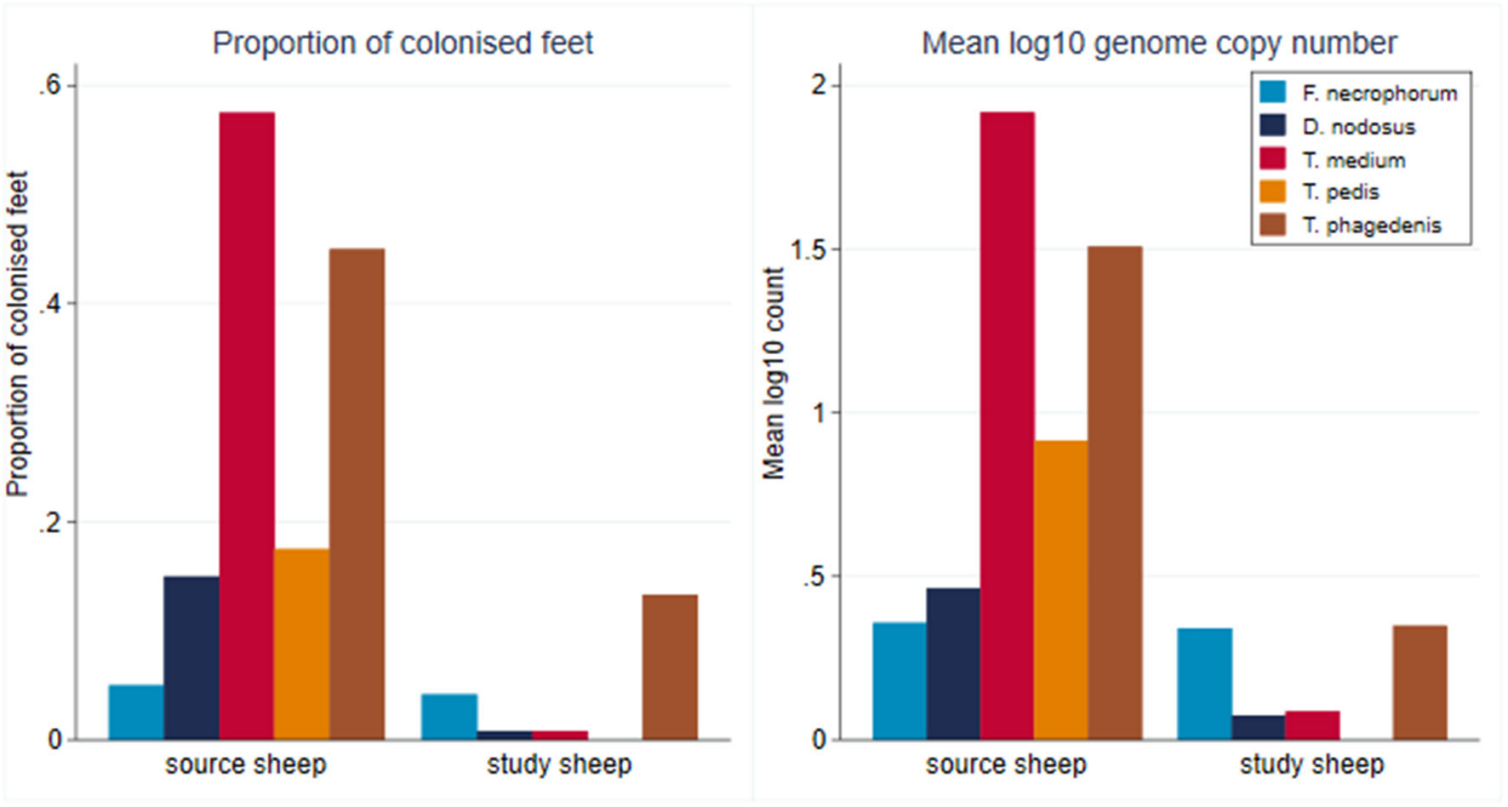
Proportion of colonized feet of study and source sheep and mean log_10_ MGCN at foot level of source and study sheep (source sheep, *n* = 10 sheep; *n* = 40 feet; study sheep *n* = 30 sheep, *n* = 120 feet).

At the start of the study, foot swab samples were collected from all the 10 source sheep (with a veterinary clinical diagnosis of CODD) and the 30 experimental study sheep (with a veterinary clinical diagnosis of healthy). All five target bacterial pathogens were present in the source sheep, confirming the introduction of these pathogens into the experimental flock as anticipated. *F. necrophorum, D. nodosus, T. medium*, and *T. phagedenis* were also detected in the healthy study sheep despite these sheep showing no clinical signs of disease in their feet (Figure 5). However, the foot level mean log_10_ MGCN for *D. nodosus, T. medium, T. pedis*, and *T. phagedenis* were all significantly lower in the study sheep compared with the source sheep, and the proportion of colonized feet was also significantly lower in the study compared to the source sheep (Table 2). The degree of colonization of the feet by *F. necrophorum* in the source and study sheep was the same (Table 2).

**Table 2:** Mean log_10_ MGCN at foot level of study sheep compared to source sheep (Student's *t*-test) (source sheep, *n* = 10 sheep, *n* = 40 feet; study sheep *n* = 30 sheep, *n* = 120 feet).

**Bacterium**	**Source sheep (*****n*** **=** **40 feet)**		**Study sheep (*****n*** **=** **120 feet)**		***P***
	**Mean log_**10**_ MGCN**	**95% CI**	**Mean log_**10**_ MGCN**	**95% CI**	
*F. necrophorum*	0.358	0.026–0.689	0.341	0.225–0.457	0.90
*D. nodosus*	0.463	0.140–0.786	0.075	0.007–0.144	<0.001
*T. medium*	1.918	1.230–2.607	0.087	0.028–0.147	<0.001
*T. pedis*	0.914	0.437–1.392	0		<0.001
*T. phagedenis*	1.508	0.959–2.056	0.350	0.263–0.436	<0.001

**Figure 6:**
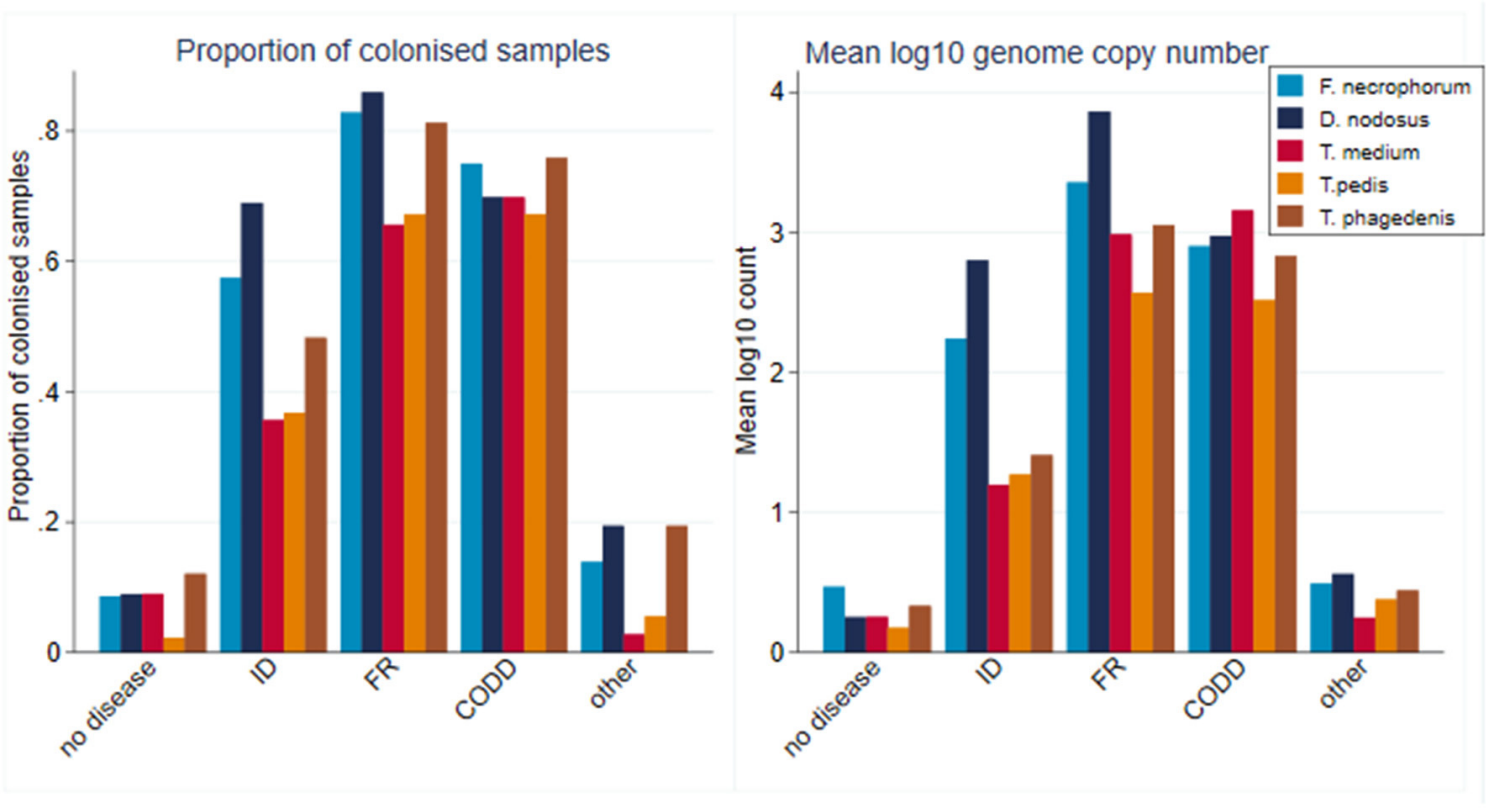
Proportion of colonized samples and mean log_10_ MGCN by recorded clinical lesion (*n* = 30 sheep, *n* = 120 feet).

A total of 2,320 samples were collected throughout the study period from the 30 sheep, with 877 samples (37.8%) yielding a positive qPCR result for at least one of the five target pathogens. There was considerable heterogenity in qPCR results, with positive results for all bacteria, namely, *F. necrophorum, D. nodosus, T. medium, T. pedis*, and *T. phagedenis* being recorded in both healthy and diseased feet, albeit with differing frequencies and pathogen loads (Figure 6). There was a tendency for both FR and CODD lesions to have higher treponeme mean log_10_ MGCN compared to ID and other lesions (Figure 6).

**Figure 7:**
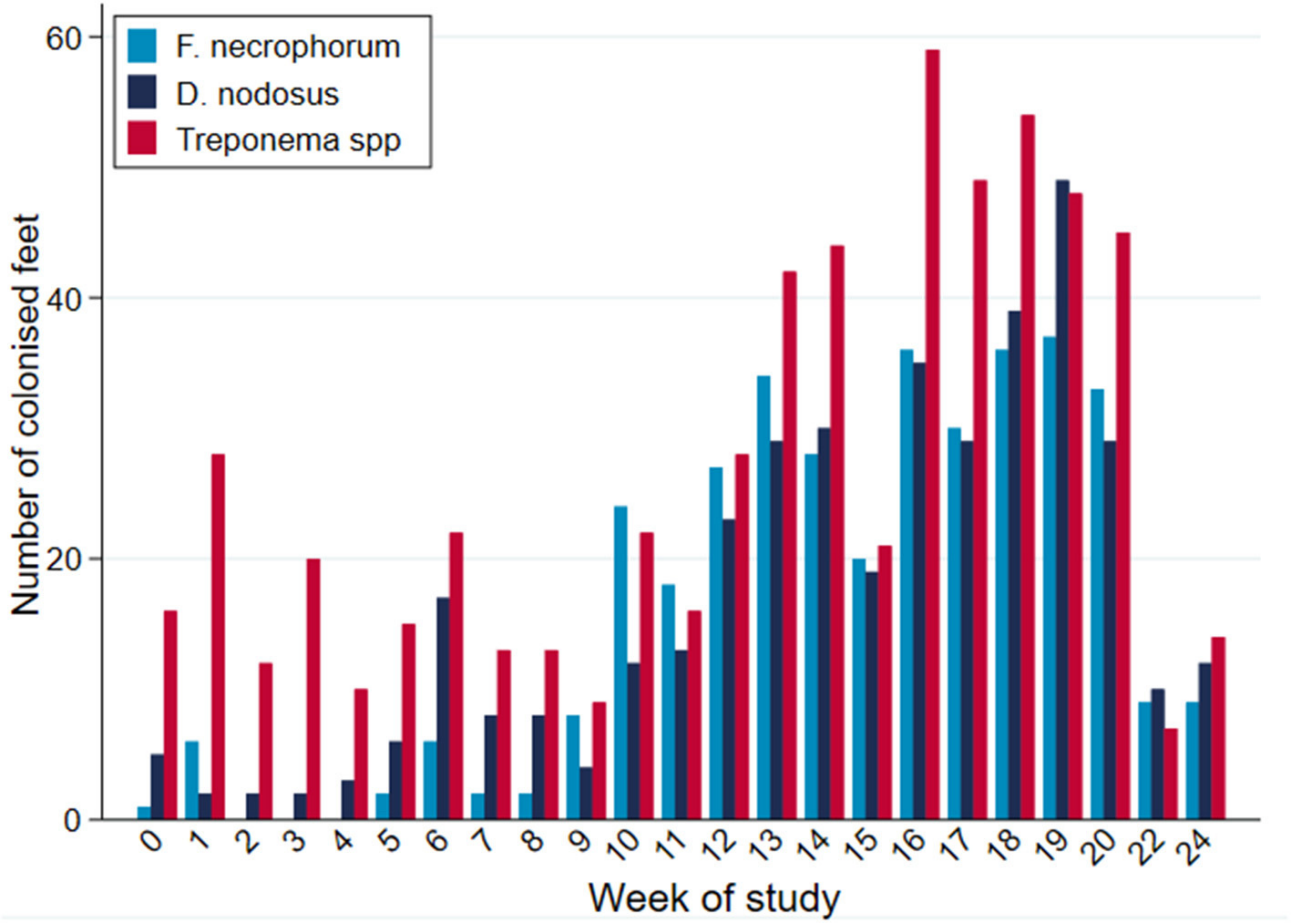
PCR-positive results, by bacterial species, per week of study period (*n* = 30 sheep, *n* = 120 feet).

While all microorganisms were detected on all sampling occasions during the study period, there were apparent broad temporal trends in detection frequency with all organisms being identified with greater frequency as the study progressed, up until commencement of treatment (Figure 7).

**Figure 8:**
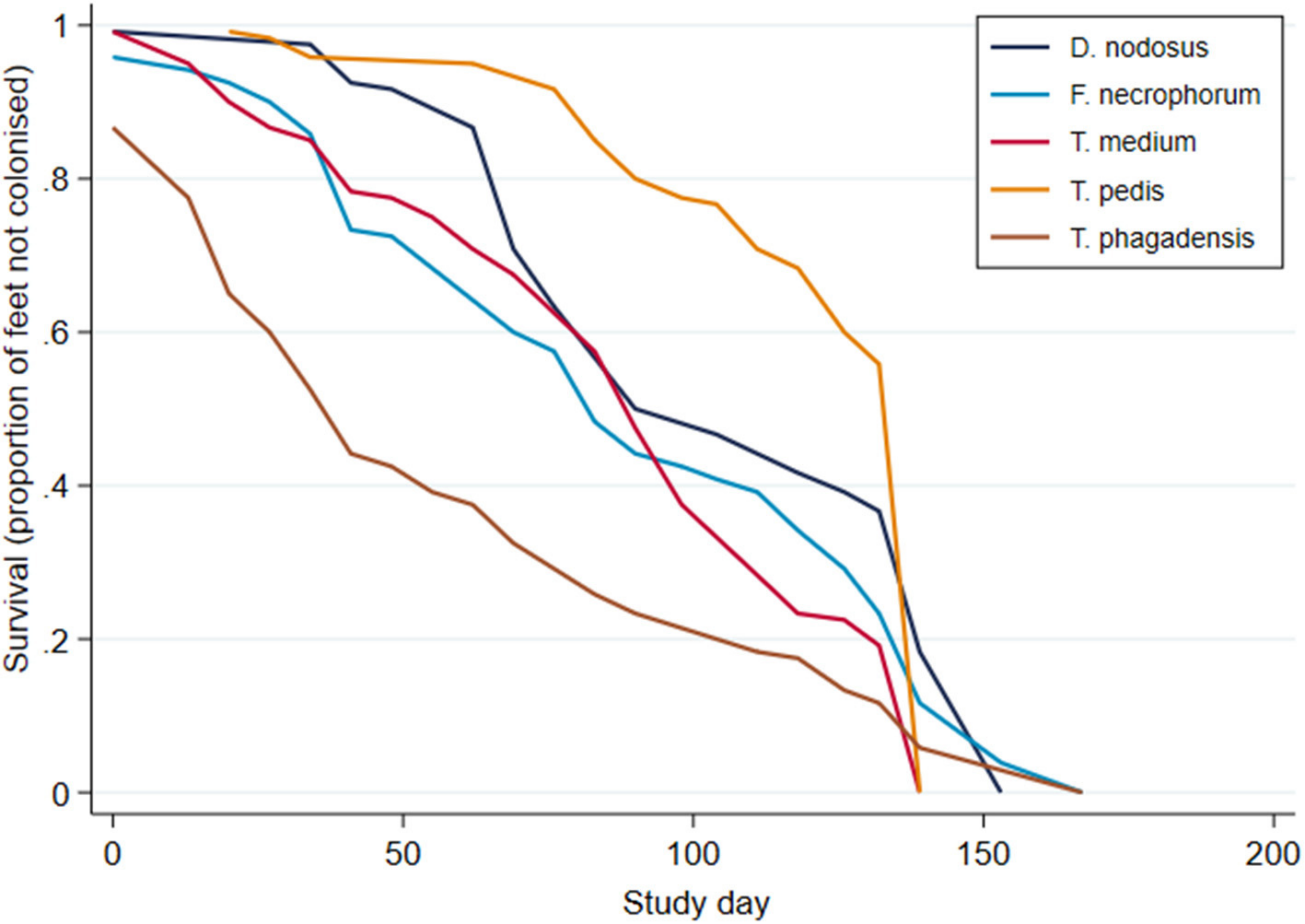
Kaplan–Meier survival curves for time to first bacterial colonization of a foot (*n* = 30 sheep, *n* = 120 feet).

Survival analysis was performed to investigate time to first identification of an organism in a foot and Kaplan–Meier survival curves plotted (Figure 8). These suggest an apparent trend for early colonization by *T. phagedenis* (median survival time, 39 days; 95% CI, 32–36 days) followed by *F. necrophorum* and *D. nodosus* (median survival time, 81 days; 95% CI, 74–88 days), *T. medium* (median survival time, 88 days; 95% CI, 81–95 days), and *T. pedis* (median survival time, 116 days; 95% CI, 95–123 days).

**Figure 9:**
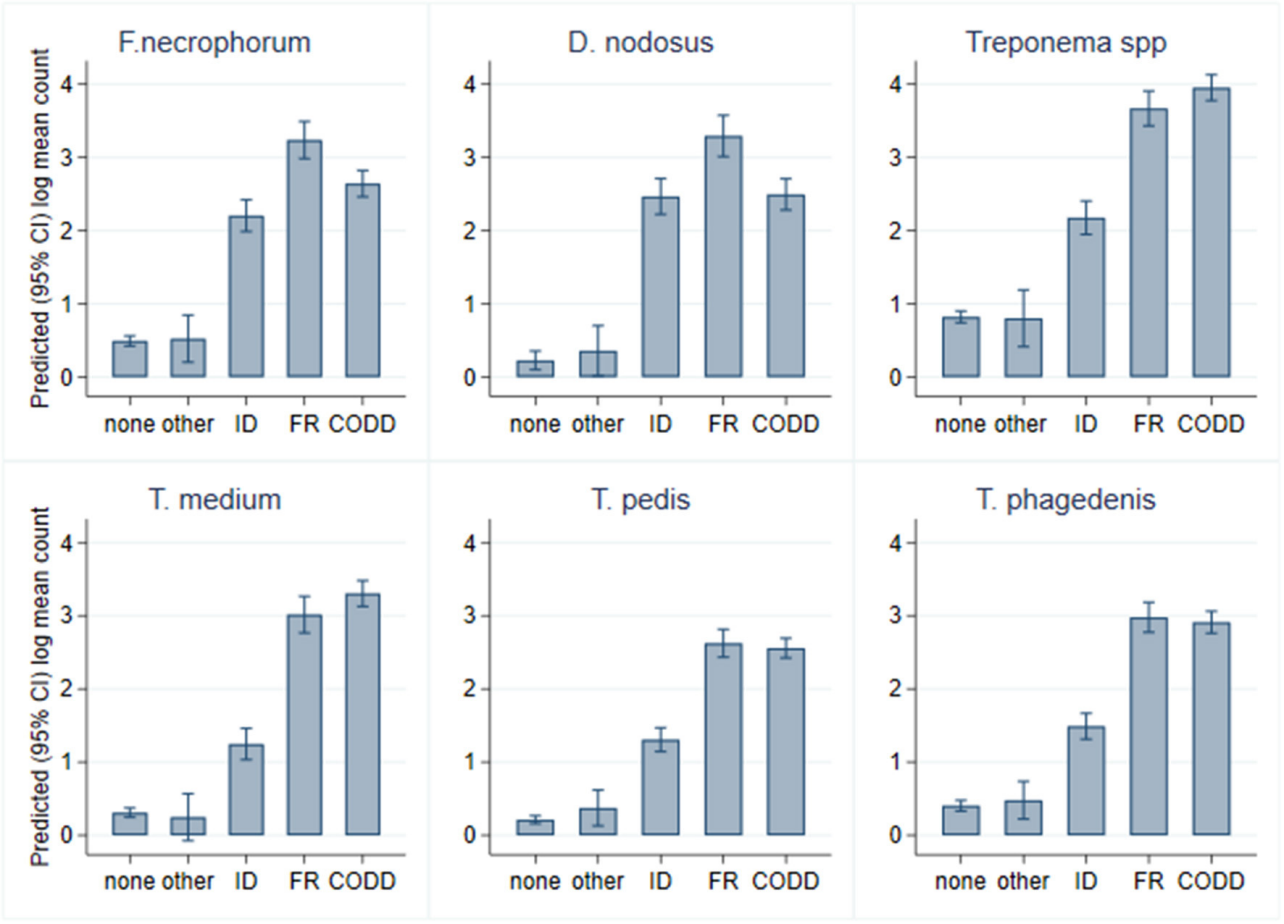
Predicted (95% CI) mean log_10_ MGCN by lesion type (*n* = 30 sheep, *n* = 120 feet).

As can be seen from Figure 6, there was considerable heterogeneity in bacterial mean log_10_ MGCN by recorded lesion type (ID, FR, and CODD). To further examine associations between each of the target bacterial species and lesion type (healthy, ID, FR, and CODD), two sets of mixed linear regression models were fitted with outcome variable being the specific bacterial species log_10_ MGCN and explanatory variables being lesion type or CODD grade. Model outputs are in Appendix 1 (Supplementary Material), and predicted marginal means estimated from the models are displayed graphically in Figures 9, **11**. While all bacterial species were present in ID, FR, and CODD, there were some trends apparent. At lesion level (Figure 9), *D. nodosus* was present at a significantly higher predicted mean log_10_ MGCN in FR lesions compared to both ID and CODD, while *Treponema* species were significantly higher in CODD and FR lesions compared to ID lesions (*p* < 0.001), although there was no statistically significant difference between predicted mean log_10_ MGCN in FR and CODD lesions (*p* = 0.47).

#### Microbiology of CODD Lesion Grades

A total of 139 samples were collected from lesions described as CODD, from 31 affected feet. As described earlier, in the case of five feet, lesions of grade 1 CODD arose *de novo*, with no prior foot disease recorded. In 26 feet, G#1 lesions were recorded, following prior lesions of ID and/or FR (progressive CODD lesions). Bacterial colonization (as a binary event) and mean bacterial log_10_ counts were compared within grade 1 lesions by origin, i.e., arising *de novo* or progressive. In the case of all bacterial species, a high proportion (>86%) of progressive G#1 lesions were colonized compared to the CODD lesions, which arose *de novo*. Furthermore, in the *de novo* CODD lesions, the qPCR signal for all bacterial species was totally absent, or insufficient as to indicate colonization, suggesting an absence of all bacterial species tested for in lesions that arose *de novo* (Table 3).

**Table 3:** Bacterial colonization of G#1 CODD lesions by previous lesion history.

**Bacterium**	**Progressive CODD lesions** **=** **46**		***De novo*** **CODD lesions** **=** **5**		***P***
	**Parameter**	**95% CI**	**Parameter**	**95% CI**	
***F. necrohporum***	
Proportion	0.913	0.792–0.976	0	0.000–0.459	<0.001
Mean log_10_ MGCN	3.65	3.27–4.02	0		<0.001
***D. nodosus***	
Proportion	0.891	0.764–0.964	0	0–0.46	<0.001
Mean log_10_ MGCN	4.06	3.58–4.54	0	0	<0.001
***T. medium***	
Proportion	0.848	0.711–0.937	0	0–0.46	<0.001
Mean log_10_ MGCN	3.87	3.33–4.42	0	0	<0.001
***T. pedis***	
Proportion	0.870	0.737–0.951	0	0–0.46	<0.001
Mean log_10_ MGCN	3.11	2.67–3.55	0.09	−0.14–0.32	<0.001
***T. phagedenis***	
Proportion	0.913	0.792–0.976	0	0–0.46	<0.001
Mean log_10_ MGCN	3.72	3.34–4.11	0.32	−0.21–0.84	<0.001

**Figure 10:**
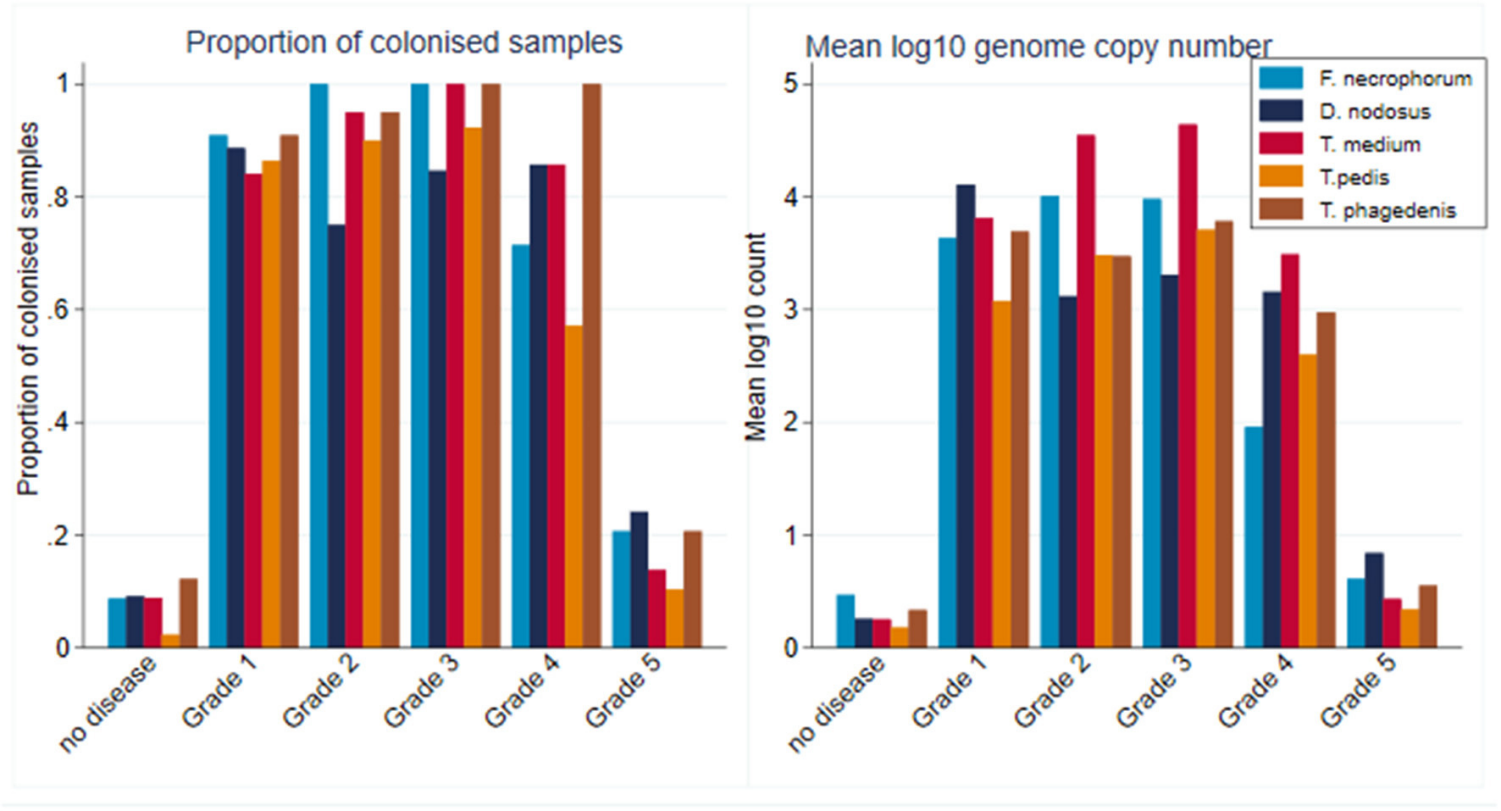
Proportion of colonized samples and mean log_10_ MGCN by CODD grade (*n* = 15 sheep, *n* = 26 feet).

Within CODD lesions, there was considerable heterogeneity in both proportion and mean log_10_ genome copy number by CODD grade (Figure 10).

**Figure 11:**
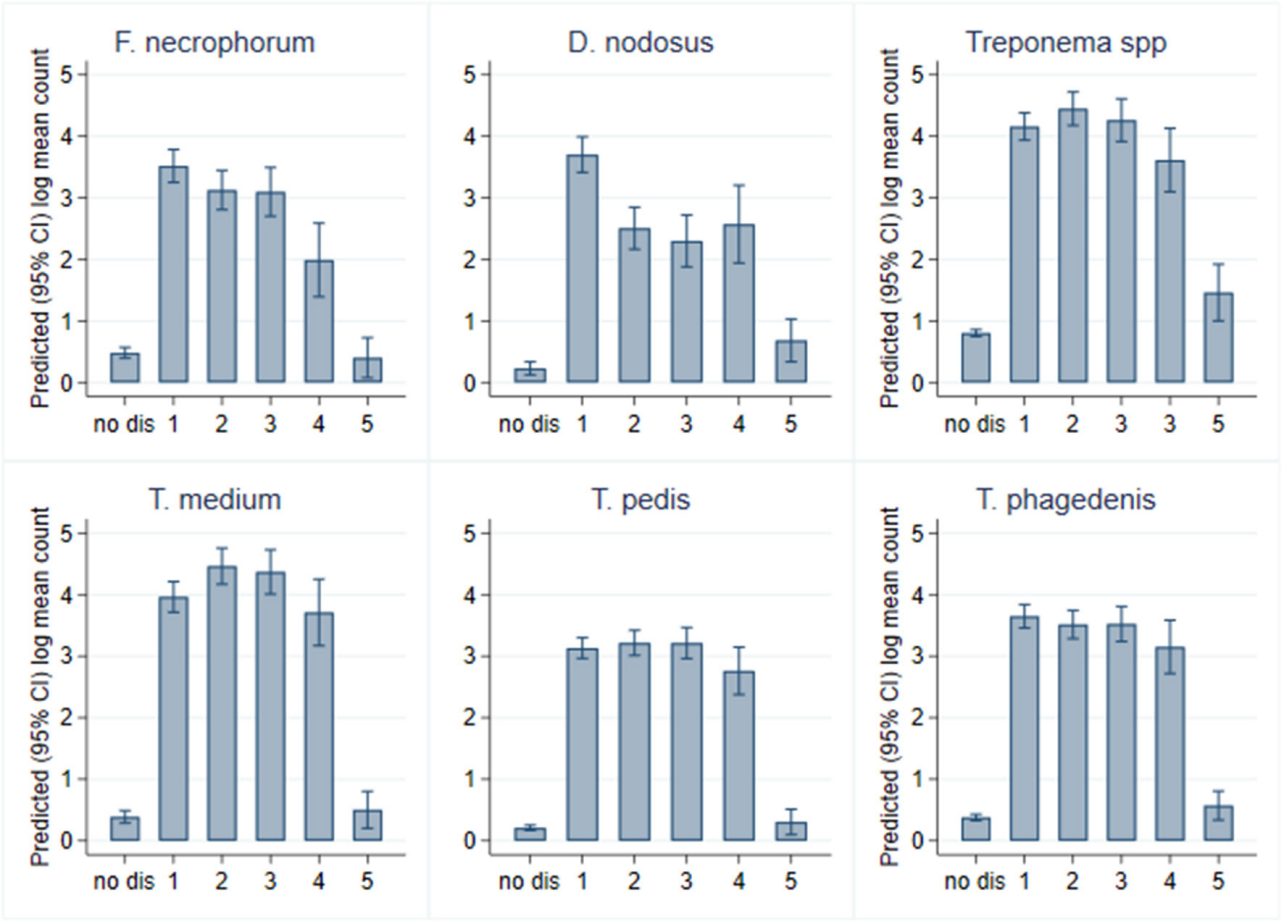
Predicted (95% CI) mean log_10_ MGCN by CODD grade (*n* = 15 sheep, *n* = 26 feet).

While all bacterial species were present in all CODD lesions irrespective of grade (Figure 11), all predicted log_10_ mean genome copy numbers were significantly lower in grade 5 lesions compared to other grades. Both predicted mean log_10_ MGCN for *F. necrophorum* and *D. nodosus* were significantly higher (*p* < 0.001) in G#1 lesions compared to all other grades. No other clear apparent trends in colonization could be observed within CODD grade.

#### Effect of Antibiotic Treatment on Microbiology of CODD Lesions

**Figure 12:**
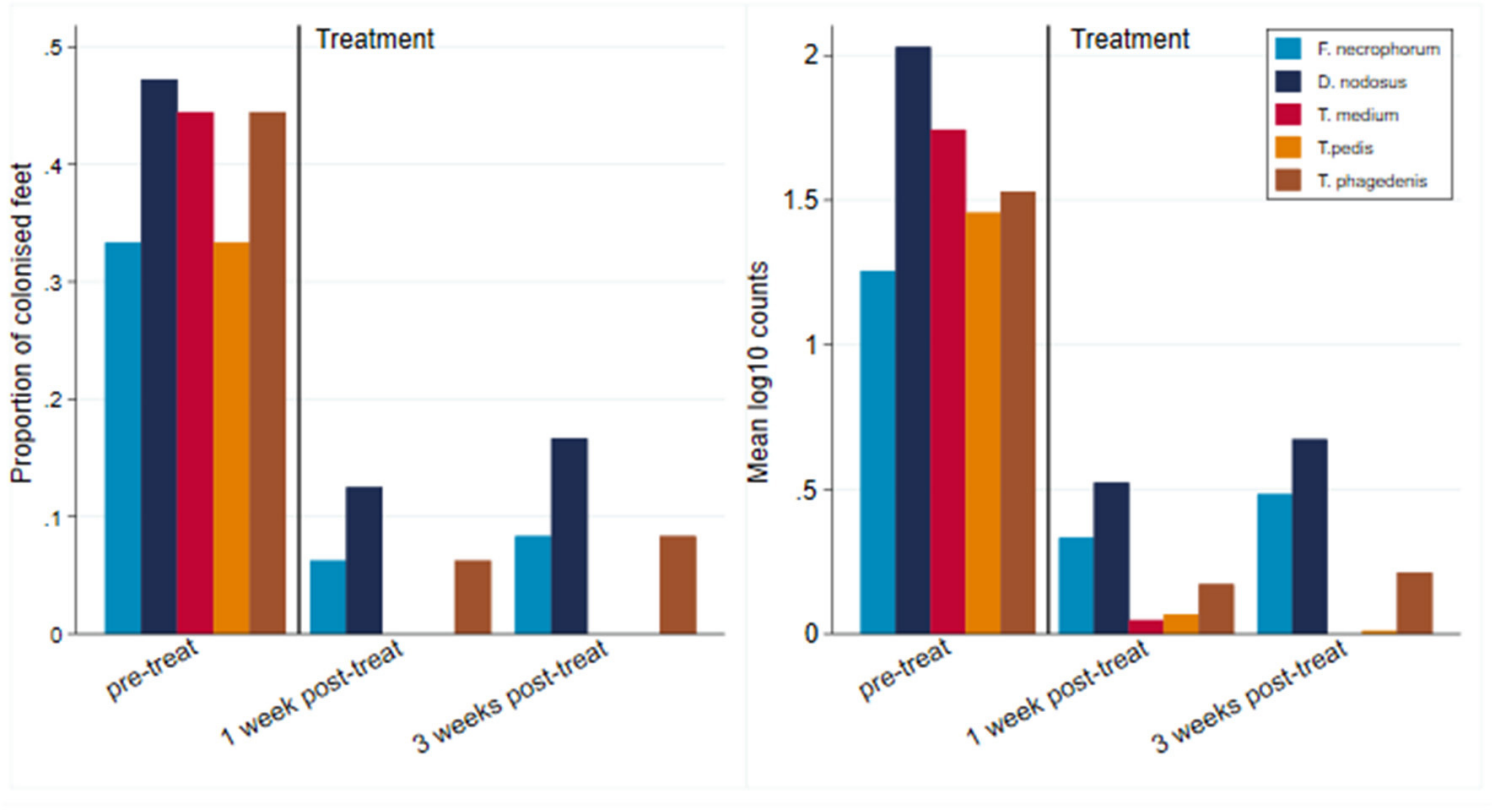
Number of colonized feet and mean log_10_ MGCN of CODD feet by pretreatment and 1 and 3 weeks post-treatment (*n* = 12 feet).

All 12 CODD lesions (G#1–G#4), present in the flock at the time of the treatment intervention, were colonized by the five target pathogens (Figure 12). One week following completion of treatment, when all of the CODD foot lesions had resolved to G#5 or healthy status, bacterial colonization and the number of colonized feet had reduced substantially in the treated animals; however, their feet remained colonized albeit at much lower levels. By 3 weeks post-treatment, when one CODD foot lesion (8.33%) had clinically recurred, the level of colonization remained at a low level and was broadly similar for *F. necrophorum, D. nodosus*, and *T. phagedenis*, while *T. medium* and *T. pedis* were now absent from the treated feet (Table 4).

**Table 4:** Percentage of feet colonized, mean (95% CI) foot level, and mean log_10_ MGCN before and after treatment (*n* = 12 feet).

**Bacterium**	**Pre-treatment**			**1 week post-treatment**			**3 weeks post-treatment**		
	**% feet**	**Mean log_**10**_ MGCN**	**95% CI**	**% feet**	**Mean log_**10**_ MGCN**	**95% CI**	**% feet**	**Mean log_**10**_ MGCN**	**95% CI**
*F. necrophorum*	33.3	1.25	0.73–1.78	6.2	0.33	0.05–0.62	8.3	0.48	0.15–0.81
*D. nodosus*	47.2	2.03	1.29–2.77	12.5	0.52	0.04–1.01	16.7	0.67	0.02–1.33
*T. medium*	44.2	1.74	1.05–2.43	0	0.05	−0.05 to 0.14	0	0.00	0.00–0.00
*T. pedis*	33.3	1.46	0.92–1.99	0	0.07	−0.01 to 0.15	0	0.01	−0.01 to 0.03
*T. phagedenis*	44.4	1.53	0.93–2.13	6.2	0.17	−0.02 to 0.37	8.3	0.21	−0.01 to 0.43

## Discussion

### Main Findings

The principle findings of the study are that CODD foot lesions can be induced experimentally in sheep by reproducing the known risk factor conditions for a naturally occurring outbreak of disease. Second, two distinct patterns of CODD lesion pathogenesis were observed. The majority of CODD lesions emerged subsequent to pre-existing ID and/or FR foot lesions in the same digit. These lesions progressed from the pre-existing ID/FR to CODD G#1 and thence through the grades to CODD G#5 and were colonized by the five target bacterial species. The second pattern was the spontaneous appearance of *de novo* G#1 CODD lesions. Here, there was no pre-existing digital pathology. This apparent G#1 lesions did not progress to CODD G#2–G#3 and were observed to spontaneously self-cure, typically within 1 week. Bacterial colonization of these lesions by the five target pathogens (as measured by qPCR) was very low or absent (log_10_ MGCN <1.303) compared to the progressive CODD lesions. This suggests that these *de novo* lesions were not, in fact, CODD but were due to other causes, e.g., trauma. This would suggest that care must be taken in ascribing such lesions to CODD in the absence of further evidence in the flock such as presence of grade 2–5 lesions. Third, the study of the microbial colonization of the different foot lesion types (ID, FR, and CODD) observed and CODD lesion grades (1–5) by the five target bacterial species showed no particular association between any individual pathogen or grouping of pathogens and a disease state. This indicates that in this experimental study, CODD had a polybacterial rather than single pathogen etiology.

#### Experimental Induction of CODD

In order to understand, as closely as possible, the etiopathogenesis of CODD as it would occur in natural field cases of the disease, the experimental study was designed to transmit and induce CODD, by mixing healthy and CODD-infected sheep in a typical, UK, indoor, sheep husbandry environment. Therefore, in this study, CODD-infected sheep were introduced to a naive flock (2); animals were housed with exposure to wet underfoot conditions (18), and there was regular close gathering of sheep for sampling (19). Transmission of CODD is hypothesized to occur between sheep indirectly through the underfoot environment or via fomites such as gloves and hoof trimming equipment (35, 36). However, to avoid cross-contamination of foot lesions for microbiological sampling, no hoof trimming was carried out in the group, and samplers' gloves were changed between handling each sheep.

The conditions described were sufficient to induce CODD lesions in 18 of the 30 sheep (60%) and 31 of the 120 feet (25.7%). The median survival time taken for the lesions to develop was 116 days (95% CI, 103–123 days). There are no other CODD experimental transmission studies for direct comparison; however, anecdotal evidence on CODD transmission in the field and experimental studies of induction of FR and BDD suggest that the time to disease induction in our study was surprisingly long. For example, in a study where BDD lesion material was directly inoculated onto abraded and wet bandaged sheep's feet (37), lesions consistent with BDD were observed after 28 days in the sheep. Experimental induction of FR in other studies report a lag of 7 days to the onset of FR when scarification of feet was combined with continued exposure to wet bedding and accumulation of fecal matter (38). Following continued exposure to a wet underfoot environment, wet bandaging of feet and direct inoculation of lesions with *D. nodosus*, FR lesions developed after 10 days (27). Similarly, recent pasture transmission studies of induction of footrot by direct inoculation of foot skin and pasture contamination with *D. nodosus* induced FR lesions in the feet typically by 8 days post-exposure (39). This compares with a median survival time (in the present study) for ID of 88 days (95% CI, 74–109 days) and for FR of 108 days (95% CI, 95–116 days). Therefore, it is apparent that foot disease induction in our experimental model was much slower than previously reported. This may be because we did not directly damage the integrity of the foot through scarification or bandaging or directly inoculate pathogens or infected material directly onto the feet. It may also reflect the time taken for the relevant pathogens in the environment to reach infective levels. It is also highly likely that the environmental conditions of the experimental model may have affected the rate of CODD disease expression in the sheep. Environmental conditions of moisture and temperature are known to affect the expression of footrot in sheep (39), and seasonal trends in field case occurrence of footrot and CODD have also been observed (18, 40). In our experimental study, the straw under the feed troughs was kept wet and the bedding deep littered; however, the sheep were not continuously exposed to wet underfoot conditions, as this would not be consistent with the management of typical UK housed sheep, which the study aimed to replicate. Environmental temperature and moisture parameters were not recorded in this study and are an area of important further work. A final reason for the slow expression of CODD in our study could be that there was no opportunity for transmission of bacterial pathogens between sheep's feet via fomites from manual handling of the feet or foot trimming equipment, which would be expected to occur in a typical farm environment.

All progressive CODD lesions (i.e., not the *de novo* ones) did develop from ID and/or FR lesions. Therefore, consistent with field evidence (2, 18, 19) and the experimental FR studies (27, 38), pre-existing damage to the foot was a prerequisite for CODD lesions to occur in this study, and CODD lesions represented the end stage of an infectious foot disease process, which began with ID/FR.

#### Phenotypic CODD Lesion Development

Two distinct patterns of CODD lesion development were observed: progressive and *de novo* CODD lesions. The majority (83%) of CODD lesions in the study were progressive CODD lesions, which developed from pre-existing ID and/or FR lesions; they progressed through stages of CODD lesions 1–5 (although there was a degree of waxing and waning of lesion grades over time), and were all heavily colonized by all five pathogens: *T. medium, T. phagedenis, T. pedis, D. nodosus*, and *F. necrophorum*. A smaller number of *de novo* CODD G#1 lesions were observed to develop from clinically healthy sheep's feet; these did not progress beyond G#1. Colonization of these *de novo* CODD lesions by the five-foot pathogens was substantially less than that observed with the progressive CODD lesions. Therefore, it is likely that in the *de novo* foot lesions, there was insufficient initial damage to the foot (by ID or FR) to allow effective colonization of the skin by the pathogenic bacteria in order for CODD lesions to fully develop.

#### Microbial Etiology of CODD Lesions

In this experiment, CODD was a disease of polymicrobial etiology. All five bacterial species, namely, *T. medium, T. phagedenis, T. pedis, D. nodosus*, and *F. necrophorum* were identified in all CODD lesion stages; however, the proportion of colonized samples and mean log_10_ MGCN of all five bacteria species were substantially lower in healthy feet, healed CODD grade 5, and treated CODD foot lesions, compared with feet affected by an active CODD lesion (grades 1–4). Thus, a clear association between bacterial colonization and foot pathology is observed, and further evidence for disease causality for the five pathogens consortium is demonstrated. This polymicrobial etiology is consistent with previous cross-sectional studies of field cases of CODD etiology where the same three DD treponeme bacteria were found in all CODD lesions; *D. nodosus* and *F. necrophorum* were also identified, but to a lesser extent (12). Metagenomic analysis of the foot microbiome during the development of CODD (from the present study) confirmed the polymicrobial etiology of CODD and clear associations between the same bacterial consortium and ID, FR, and CODD (28).

While it is clear that CODD is polymicrobial in nature, there were apparent trends in the data regarding the temporal development of CODD. As foot pathology progressed from ID to FR to CODD (grades 1–4), linear regression models exploring bacterial colonization suggested that *D. nodosus* was present at significantly higher mean log_10_ concentrations in FR lesions compared to both ID and CODD (Figure 9); the treponeme bacteria colonized FR and CODD lesions at a higher mean log_10_ concentrations compared to the ID lesions (Figure 9); and finally, *D. nodosus* and *F. necrophorum* counts were significantly higher (*p* < 0.001) in the earlier G#1 CODD lesions compared to the later ones (G#2–G#4) (Figures 9, 11). The survival analysis is not entirely consistent with the modeling data, as early colonization by *T. phagedenis* is then followed by *F. necrophorum* and *D. nodosus*, and then colonization by *T. medium* and *T. pedis* is observed.

In the pathogenesis of FR, the strain of *D. nodosus* present affects the ability of the bacteria to invade the epidermis and cause clinical disease (due to the expression of the AprV_2_ gene) (41). In the current study, the strain of *D. nodosus* was not examined, but now that we have shown a clear link in the pathogenesis of FR and CODD, this is an important area of future work.

Biosecurity measures and practices to prevent introduction of disease-causing agents into a population are an essential component of almost all infectious disease control strategies. The findings of the current study have important implications for flock biosecurity. The current advice for the prevention of the introduction of CODD into naive flocks is that all feet of all brought sheep should be examined upon arrival at the farm for CODD lesions (42). However, this may not be sufficient alone to prevent the introduction of CODD. At the start of the study, there was evidence of low-level colonization of the healthy experimental study sheep's feet with *T. medium* (0.83% of feet), *T. phagedenis* (13.3% of feet)*, D. nodosus* (0.83% of feet), and *F. necrophorum* (4.17% of feet) (but not *T. pedis*). The flock, from which the sheep were sourced, did have a history of infection with ID and FR but had never observed CODD. It would be expected to find low-level colonization with *F. necrophorum* in healthy sheep's feet and *D. nodosus*, as reported in studies of FR in sheep (43). However, it was surprising to find low levels of CODD- and DD-associated treponemal species in the healthy feet from a farm with no known history of CODD in sheep or DD in the beef cattle on the farm. This contrasts with previous reports, employing routine gel-based PCR techniques, which demonstrated an absence of CODD/BDD treponemes in healthy vs. infected CODD foot biopsy tissue (12). The present study offers no evidence that the bacterial load detected in healthy feet is sufficient to initiate infection or was viable or may even be an artifact associated with the chosen qPCR cutoff values. Alternatively, given that *T. phagedenis* has been previously reported as a commensal of the GI tract in humans, chimpanzees (44), and healthy cattle (45), its presence on healthy feet could in fact represent commensal contamination from the GI tract and not be associated with foot disease *per se*. Indeed, recent work comparing bovine DD and human non-pathogenic *T. phagedenis* demonstrates the presence of unique gene clusters encoding key survival factors and a putative secretion system within the disease-associated bovine *T. phagedenis* strains (46). Using qPCR assays to target such virulence factors may enable better pathogen load determination to better describe disease progression in the future. However, based on the current data we have presented here, while the current advice regarding the examination of sheep feet at entry should serve to reduce the risk of introducing CODD—given that diseased sheep have a much greater bacterial load—it may not totally eliminate all risk.

#### Effect of Treatment on CODD Lesions

There was 100% clinical cure rate of CODD lesions (CODD G#5), observed 1 week after the treatment intervention of two doses of long-acting amoxicillin and environmental decontamination. However, one lesion (8.3%) was observed to recur 2 weeks later. The microbiology of the feet also clearly demonstrates substantial reduction in the mean log_10_ MGCN of all five bacterial species 1 and 3 weeks post-treatment. Importantly, bacterial cure was not established in all feet. Of all feet, 16.7% remained colonized by *D. nodosus* and 8.3% by *F. necrophorum* and *T. phagedenis*, although bacteriological cure was achieved for *T. pedis* and *medium*. The antibiotic therapy chosen (amoxicillin) was selected based on its previously reported field clinical cure rates for CODD (71%) (19) and *in vitro* bacterial sensitivity assays for treponeme bacteria (47). Therefore, the results of the current study are consistent with these data and emphasize that treated animals may still be infectious to the flock.

#### Study Limitations

The principal limitation of the study is that is an experimental study, and the results require validation in the field. However, every effort was made to mimic known field conditions, and the data are consistent with previous field studies. Furthermore, a dual sampling strategy of swabs and different biopsy depths in the field together with fluorescent *in situ* hybridization of key pathogens may help confirm and dissect findings in the future. Here, we used housekeeping genes as targets of qPCR, a well-established methodology; however, in future studies, if these were to be supplemented with qPCR assays targeting recently identified pathogenic determinants, this may enable better associations of pathogen presence with disease progression.

## Conclusions

1. This study presents a new understanding of the etiopathogenesis CODD in sheep, whereby the majority of CODD lesions developed from pre-existing ID/and or FR lesions.
2. CODD has a polymicrobial etiology associated with infection with the bacteria *T. medium, T. phagedenis, T. pedis, D. nodosus*, and *F. necrophorum*.
3. As foot pathology progressed chronologically from ID to FR to CODD (grades 1–4), *D. nodosus* was found to be highest concentrations in FR lesions compared with ID and CODD lesions, while *T. medium, T. phagedenis*, and *T. pedis* were present in higher concentrations in the later stages of lesions (FR and CODD).
4. Of the clinical cure rate of CODD lesions, 91.7% was achieved with the amoxicillin-based treatment intervention by 3 weeks post-treatment; however, some feet remained colonized at a low level by *D. nodosus, F. necrophorum*, and *T. phagedenis*.
5. Healthy, healed CODD, and antibiotic-treated CODD feet may all be colonized by one or more of the five pathogens associated with CODD infection, namely, *T. medium, T. phagedenis, T. pedis, D. nodosus*, and *F. necrophorum*, and could therefore be a source of infection for between and within flock CODD disease spread.

## Data Availability Statement

The original contributions presented in the study are included in the article/Supplementary Material, further inquiries can be directed to the corresponding author/s.

## Ethics Statement

This animal study was reviewed and approved by Home Office Project License PPL 708756 and University of Liverpool Ethics VREC417.

## Author Contributions

JD, DG-W, NE, and SDC co-designed, obtained financial support, and co-supervised the study. JA, JD, and SRC collected the samples. SRC, GS, and NE performed laboratory analyses. DG-W performed data analysis. JD wrote the manuscript. GS, JA, SRC, NE, SDC, and DG-W critically evaluated the manuscript. All authors contributed to the article and approved the submitted version.

## Funding

This work was funded by a Biotechnology and Biological Sciences Research Council (BBSRC) Industrial Partnership Award (IPA) Research Grant (BB/N002121/1) partnering Hybu Cig Cymru HCC/Meat Promotion Wales and AHDB Beef and Lamb (a division of the Agriculture and Horticulture Development Board, UK).

## Conflict of Interest

JA is currently employed by Wern Vets CYF, at the time of carrying out the research he was employed by the University of Liverpool. The remaining authors declare that the research was conducted in the absence of any commercial or financial relationships that could be construed as a potential conflict of interest.

## Publisher's Note

All claims expressed in this article are solely those of the authors and do not necessarily represent those of their affiliated organizations, or those of the publisher, the editors and the reviewers. Any product that may be evaluated in this article, or claim that may be made by its manufacturer, is not guaranteed or endorsed by the publisher.

## Abbreviations

CODD: contagious ovine digital dermatitis
FR: footrot
ID: interdigital dermatitis
UK: United Kingdom of Great Britain and Northern Ireland
qPCR: quantitative polymerase chain reaction
MGCN: mean genome copy number
ARRIVE: Animal Research: Reporting of *In Vivo* Experiments.
